# Diversification dynamics of rhynchostomatian ciliates: the impact of seven intrinsic traits on speciation and extinction in a microbial group

**DOI:** 10.1038/s41598-017-09472-y

**Published:** 2017-08-30

**Authors:** Peter Vďačný, Ľubomír Rajter, Shahed Uddin Ahmed Shazib, Seok Won Jang, Mann Kyoon Shin

**Affiliations:** 1 Department of Zoology, Comenius University in Bratislava, Bratislava, 84215 Slovak Republic; 2 Department of Biological Sciences, University of Ulsan, Ulsan, 44610 Korea

## Abstract

Ciliates are a suitable microbial model to investigate trait-dependent diversification because of their comparatively complex morphology and high diversity. We examined the impact of seven intrinsic traits on speciation, extinction, and net-diversification of rhynchostomatians, a group of comparatively large, predatory ciliates with proboscis carrying a dorsal brush (sensoric structure) and toxicysts (organelles used to kill the prey). Bayesian estimates under the binary-state speciation and extinction model indicate that two types of extrusomes and two-rowed dorsal brush raise diversification through decreasing extinction. On the other hand, the higher number of contractile vacuoles and their dorsal location likely increase diversification via elevating speciation rate. Particular nuclear characteristics, however, do not significantly differ in their diversification rates and hence lineages with various macronuclear patterns and number of micronuclei have similar probabilities to generate new species. Likelihood-based quantitative state diversification analyses suggest that rhynchostomatians conform to Cope’s rule in that their diversity linearly grows with increasing body length and relative length of the proboscis. Comparison with other litostomatean ciliates indicates that rhynchostomatians are not among the cladogenically most successful lineages and their survival over several hundred million years could be associated with their comparatively large and complex bodies that reduce the risk of extinction.

## Introduction

Diversity is a phenomenon of life. The asymmetry in the ability of lineages to diversify has become one of the central objectives in trait-dependent diversification studies^1–4^. It has been shown that large differences in species numbers between lineages might have originated in two ways, through increasing the speciation rate or decreasing the extinction rate. The high speciation rates cause a direct rise in the number of lineages, while the low extinction rates result in a gradual accumulation of lineages^4–6^. The cladogenic success is also connected with evolution of uniquely beneficial character states, so-called key innovations. Their detection is often associated with a quest for pronounced asymmetry in character state transition rates, i.e., convergent evolution when traits have appeared many times but have been rarely lost^7, 8^.

Diversification dynamics is an understudied field in microbial groups. Ciliates seem to be a suitable microbial model to explore trait-dependent diversification, because they are both species-rich and comparatively morphologically complex^9, 10^. These eukaryotic microbes are also diverse functionally, acting in a variety of roles such as fine to coarse filter feeders, hunters, scrapers or sucking feeders^11, 12^. Within the twelve recognised ciliate classes, the predatory way of life is mostly confined to the Litostomatea and the Prostomatea^10^. A morphologically very distinct group, the Rhynchostomatia, arose within the Litostomatea during the Palaeozoic period^13^. Rhynchostomatians are large to gigantic predatory ciliates, being from 100 μm up to 1,500 μm long^14^! Their raptorial lifestyle is uniquely associated with a conspicuous proboscis that carries batteries of toxicysts, which are extrusive organelles used to kill the prey, and a dorsal brush, which is a sensoric structure possibly involved in prey detection. Interestingly, rhynchostomatians are divided into two fundamental monophyletic groups with highly uneven distribution of taxa: the Tracheliida with just two species and the Dileptida with 64 species and subspecies^14^.

Using a range of statistical phylogenetic methods, we assessed clade-specific rates and searched for shifts in diversification rates during the evolutionary history of rhynchostomatians. Further, we investigated the impact of key quantitative and qualitative morphological features on speciation, extinction, and net-diversification rates of rhynchostomatian lineages. This complex approach enabled us to recognise which intrinsic traits increase diversification directly through elevating speciation rate, which indirectly via decreasing extinction rate, and which intrinsic traits do not cause asymmetries in cladogenesis.

## Results

**Figure 1:**
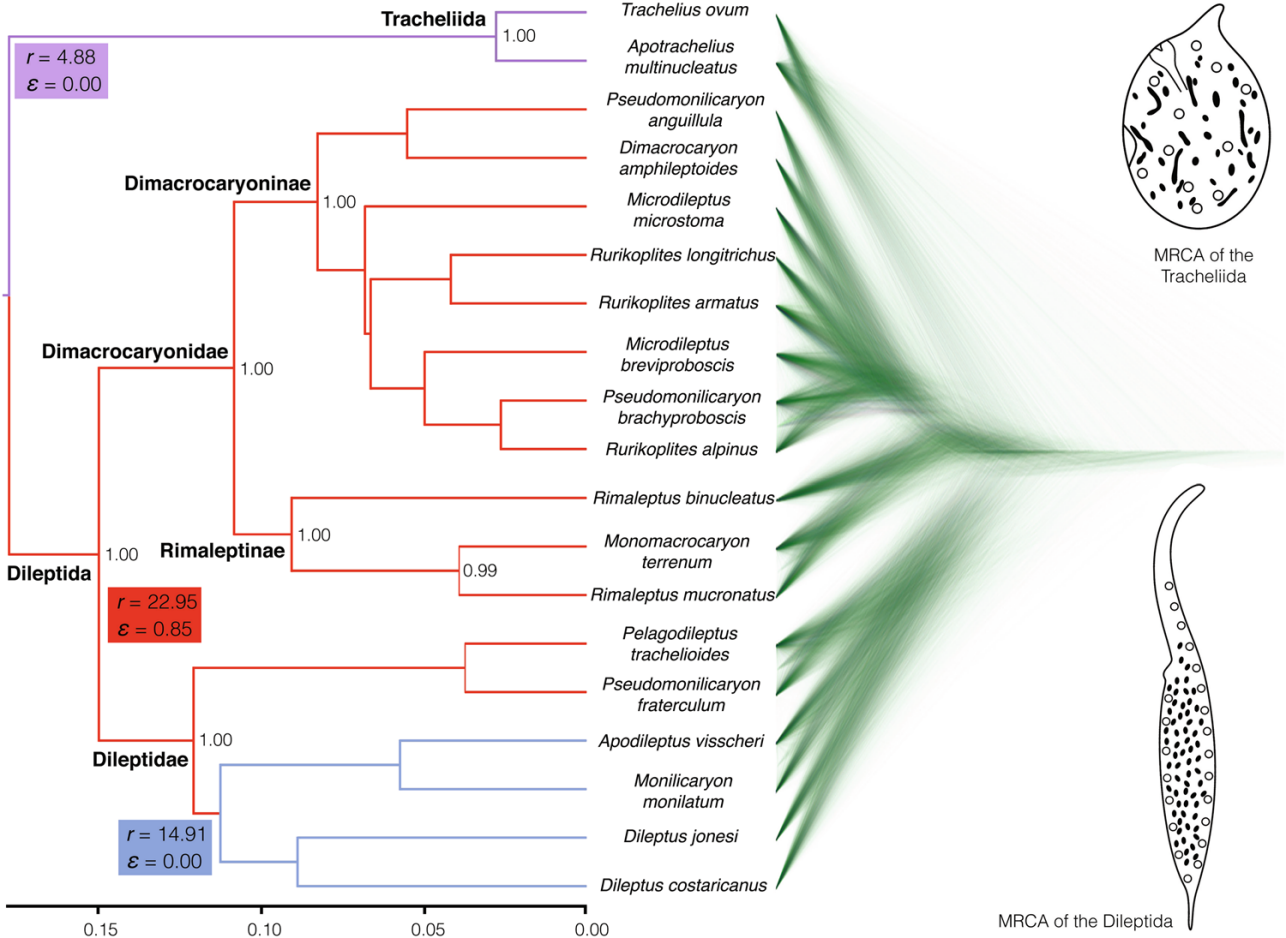
Maximum clade credibility species tree and corresponding cloudogram of the subclass Rhynchostomatia. The cloudogram is shown in green colour and consists of 7,500 superimposed post burn-in trees from the posterior distribution of the BEAST coalescent analysis. Branch colours designate tree partitions inferred to evolve at three significantly different rates by MEDUSA, with maximum-likelihood estimates of net-diversification rate (*r*) and extinction fraction (*ε*) for each tree partition. The tracheliid lineage diversified at a net-diversification rate of 4.88 (violet colour), while the dileptid clade at a net-diversification rate of 22.95 with an extinction fraction of 0.85 (red colour). However, there was a statistically significant shift in net-diversification rate to 14.91 within dileptids at the node uniting members with predominantly scattered macronuclear nodules (blue colour). The scale bar indicates number of substitutions. Only posterior probabilities > 0.95 are shown.

Concatenated and coalescent-based Bayesian phylogenetic analyses of the 18 S rRNA gene, ITS region, and first two domains of the 28 S rRNA gene brought similar results, consistent with previous studies^15–19^. The subclass Rhynchostomatia was divided into two fundamental lineages, the order Tracheliida and the order Dileptida, whose monophylies were strongly statistically supported by both analyses. Likewise, monophylies of the families Dileptidae and Dimacrocaryonidae were fully statistically supported. As concerns dimacrocaryonids, the subfamily Dimacrocaryoninae consistently received full support, while the subfamily Rimaleptinae was either strongly supported or was depicted as paraphyletic containing the former subfamily (Fig. 1; Supplementary Fig. S1). Since the internal relationships within the three main dileptid lineages were rather poorly resolved, the majority of analyses was performed on a set of 100 randomly selected post-burn-in trees from the coalescent inference.

As concerns diversification of the subclass Rhynchostomatia, a birth-death model was favoured over a pure-birth model (BayesFactor BF = 28.24). Analyses of clade specific rates under the birth-death model indicated that the three main dileptid lineages (Dileptidae, Dimacrocaryoninae, and Rimaleptinae) diversified at a similar rate that was higher than in the tracheliid lineage (Supplementary Table S1). A similar pattern was also recognised by MEDUSA in that the tracheliid lineage diversified at a lower rate (*r* = 4.8780, yule model, AICc = 41.3196) than the dileptid clade (*r = *22.9523, ε = 0.84624, birth-death model, AICc = 34.7045). MEDUSA also indicated that there was a statistically significant shift in diversification rate within the family Dileptidae, i.e., at the node uniting members with predominantly scattered macronuclear nodules (*r* = 14.9072, yule model, AICc = 37.1112) (Fig. 1).

**Figure 2:**
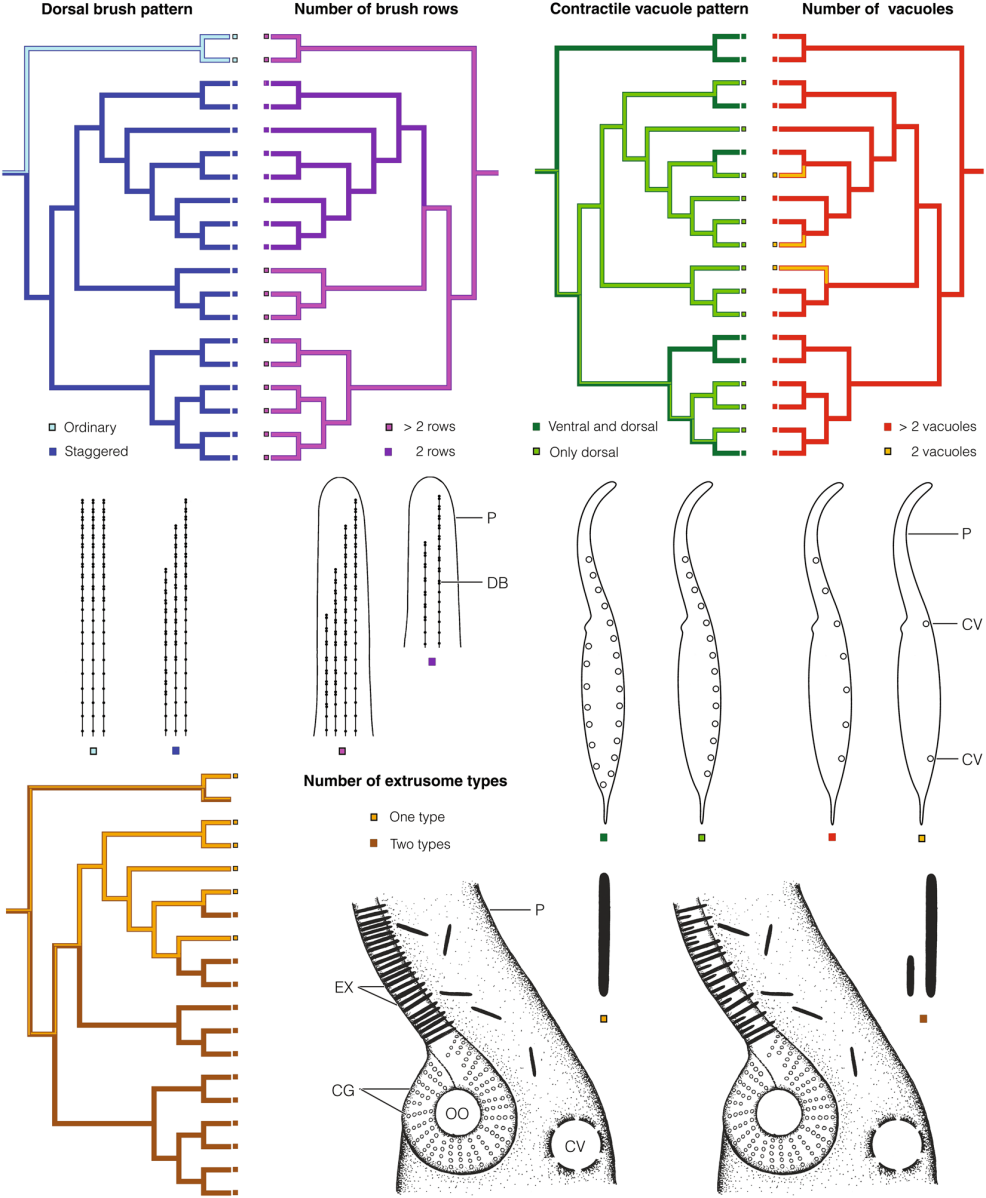
Maximum-parsimony character state reconstruction of five binary traits. The maximum clade credibility tree shown in Fig. 1 served as a scaffold for reconstruction of character state evolution. Squares at tips of branches show the character state of each taxon. For further details, see Supplementary Table S2. Explanations: CG, cortical granules; CV, contractile vacuoles; DB, dorsal brush; EX, extrusomes; P, proboscis.

**Figure 3:**
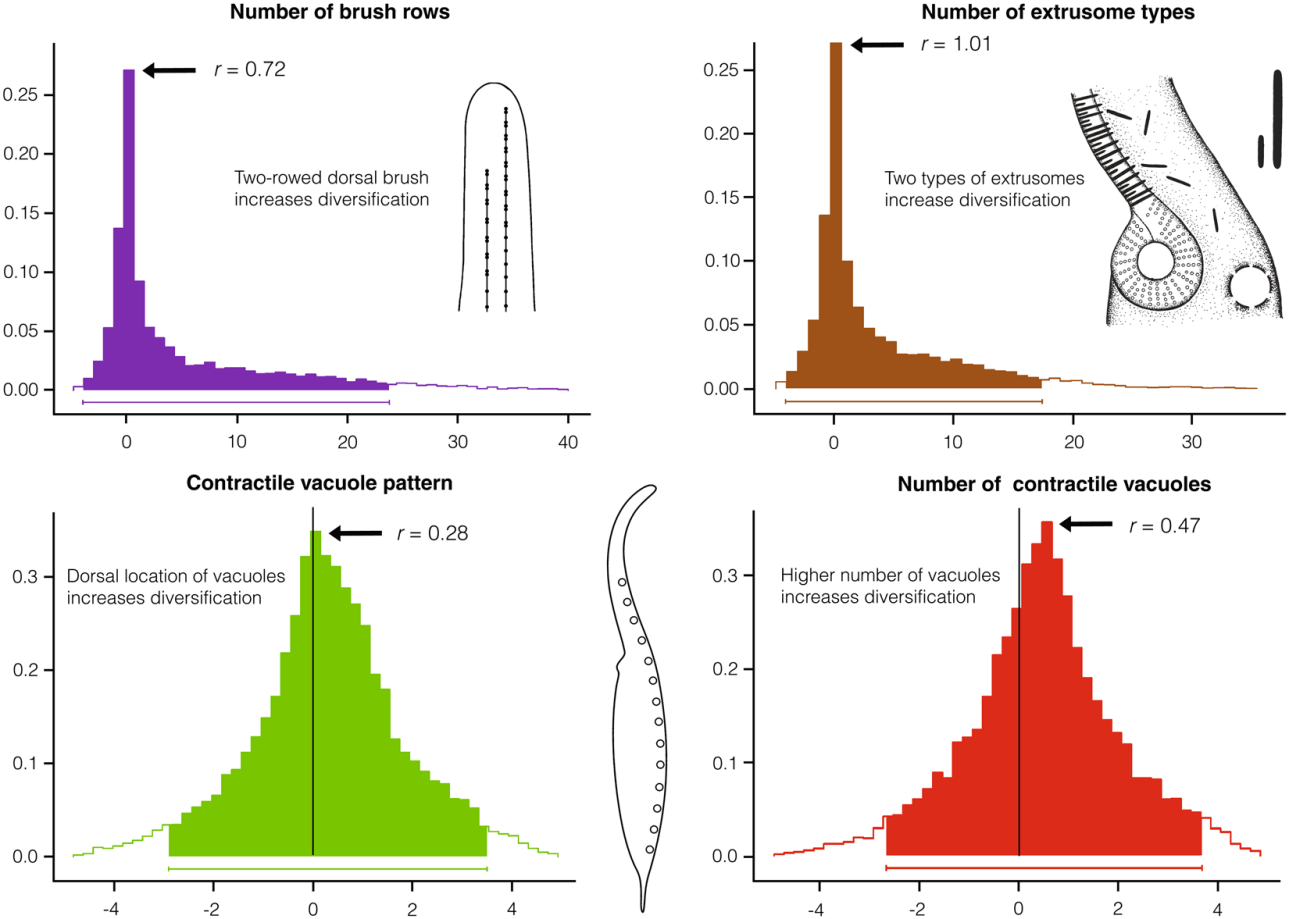
Posterior densities of differences in net-diversification rates between lineages with two-rowed and multi-rowed dorsal brush, between lineages with two types and one type of extrusomes, between lineages with dorsally and dorsally as well as ventrally located contractile vacuoles, and between lineages with more then two and two vacuoles. The x-axis represents the difference in net-diversification rates (∆*r*) between lineages with particular character states (∆*r* = *r*_1_ − *r*_0_; subscripts indicate different character states as specified in Supplementary Table S8). The ∆*r* distribution was obtained from MCMC simulations on a sample of 100 trees from the posterior distribution of the BEAST coalescent analysis. The y-axis represents posterior densities of differences in net-diversification rates, as calculated on the basis of the MCMC simulations. Arrows denote median of the difference in net-diversification rates (∆*r*). For interquartile ranges and further details, see Table 1. Ninety-five per cent intervals of highest posterior density are indicated below densities.

According to reconstruction analyses, the most recent common ancestor (MRCA) of the order Tracheliida had an ordinary dorsal brush, i.e., all brush rows started at the top of the proboscis. In contrast, the MRCA of the order Dileptida possessed a staggered dorsal brush, i.e., brush rows were arranged in a step-like fashion (Fig. 2; Supplementary Table S2). Since there is a dramatic asymmetry in the number of taxa between these two orders (2 vs. 64), we speculate that transformation of this sensoric structure into a staggered pattern could be associated with the cladogenic success of the dileptid lineage. A further change in the dorsal brush structure happened in the MRCA of the subfamily Dimacrocaryoninae, when a two-rowed brush evolved (Fig. 2; Supplementary Table S2). The number of brush rows is also connected with a rather pronounced asymmetry, i.e., only about 35% of rhynchostomatian taxa have two-rowed brush while about 65% of rhynchostomatians still display more than two rows. Bayesian estimates under the binary-state speciation and extinction (BiSSE) model indicate that two-rowed dorsal brush is associated with reduced extinction rate (Table 1; Supplementary Table S3). Differences in net-diversification rates between two-rowed and multi-rowed lineages in MCMC simulations are distinctly shifted to the positive side of the x-axis (Fig. 3), indicating that lineages with two-rowed brush have a lower risk of extinction.

**Table 1:** Parameterisation of best fitting BayesRate models for binary state diversification of rhynchostomatian ciliates. Tabulated are median rates of speciation (*λ*_0_, *λ*_1_) and extinction (*μ*_0_, *μ*_1_) as well as net-diversification rates (*r*_0_ = *λ*_0_ − *μ*_0_, *r*_1_ = *λ*_1_ − *μ*_1_) and their differences (∆*r* = *r*_1_ − *r*_0_) ± interquartile range estimated over 100 trees from the posterior distribution of the BEAST coalescent analysis during MCMC simulations in BayesRate.

Characteristic	Speciation	Extinction	*λ*_0_	*λ*_1_	*μ*_0_	*μ*_1_	*r*_0_	*r*_1_	∆*r*
Number of dorsal brush rows	constrained	free	6.61 ± 16.95	6.61 ± 16.95	2.15 ± 5.94	1.51 ± 1.62	3.13 ± 9.82	1.12 ± 8.63	0.72 ± 0.48
Number of extrusome types	constrained	free	6.27 ± 13.88	6.27 ± 13.88	4.00 ± 13.30	1.99 ± 5.39	1.55 ± 1.72	3.00 ± 7.59	1.01 ± 6.49
Number of contractile vacuoles	free	free	2.03 ± 4.09	6.14 ± 17.85	0.68 ± 2.43	3.79 ± 17.06	1.01 ± 1.61	1.55 ± 1.82	0.47 ± 1.78
Contractile vacuole pattern	free	free	2.71 ± 4.79	5.80 ± 20.43	1.07 ± 3.55	3.54 ± 19.53	1.17 ± 1.47	1.54 ± 1.67	0.28 ± 1.71
Number of micronuclei	constrained	constrained	5.68 ± 16.17	5.68 ± 16.17	3.45 ± 15.58	3.45 ± 15.58	1.54 ± 1.65	1.54 ± 1.65	0.00 ± 0.00

The obtained posterior densities and distribution of differences in net-diversification rates (∆*r*) are shown in Fig. 3. Subscripts indicate character states as coded in Supplementary Table S8. For further details, see Supplementary Table S3.

As concerns the number of extrusome types, reconstruction analyses suggest that the MRCAs of the order Tracheliida and the subfamily Dimacrocaryoninae had a single type of extrusomes anchored in the proboscis oral bulge, while the MRCAs of the family Dileptidae and the subfamily Rimaleptinae displayed two extrusome types (Fig. 2; Supplementary Table S2). The Bayesian BiSSE analyses indicate that two types of extrusomes are, similarly as the two-rowed dorsal brush, associated with reduced extinction rate (Table 1; Supplementary Table S3). This is also indirectly corroborated in that 76% of extant rhynchostomatian taxa possess two types of extrusomes. Differences in net-diversification rates between lineages with one type and two types of extrusomes in MCMC simulations are also conspicuously shifted towards the positive side of the x-axis (Fig. 3), suggesting significant difference in their rates.

The MRCAs of the order Tracheliida and the family Dileptidae possibly had many contractile vacuoles arranged both ventrally and dorsally. In contrast, the MRCAs of the subfamilies Dimacrocaryoninae and Rimaleptinae possessed two dorsally arranged vacuoles (Fig. 2; Supplementary Table S2). BiSSE indicates that the higher number of contractile vacuoles and their dorsal location increase diversification rate on average (Table 1, Fig. 3; Supplementary Table S3). This is also indirectly sustained in that 85% of taxa have more than two contractile vacuoles, and these are arranged dorsally in 72% of taxa. Although intervals of differences in net-diversification rates in MCMC simulations exceed zero towards the negative side of the x-axis (Fig. 3), mean values are significantly positive for both, the contractile vacuole pattern (t = 19.22, df = 9999, p < 2.2e-16, 95% confidence interval of mean 0.26–0.32) and the number of vacuoles (t = 28.90, df = 9999, p < 2.2e-16, 95% confidence interval of mean 0.43–0.49). Thus, Bayesian MCMC analyses suggest a trend that the higher number of contractile vacuoles and their dorsal location are associated with increasing diversification.

**Figure 4:**
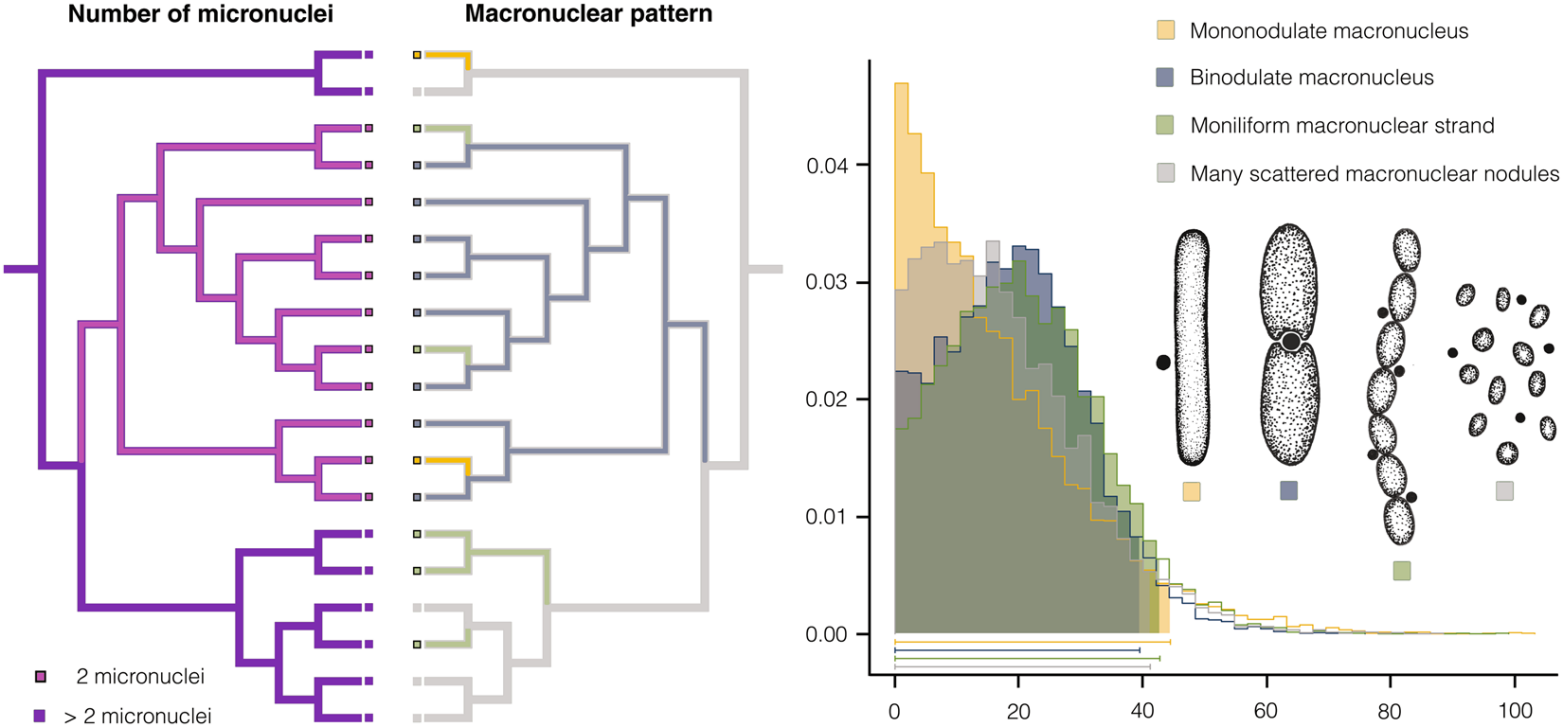
Maximum-parsimony character state reconstruction of micro- and macronuclear traits as well as posterior densities of speciation rates for four macronuclear patterns in Bayesian MuSSE analyses. The maximum clade credibility tree shown in Fig. 1 served as a scaffold for both reconstruction analyses. Squares at tips of branches show the character state of each taxon. The x-axis represents distribution of speciation rates for mononodulate macronucleus (yellow colour), binodulate macronucleus (blue colour), moniliform macronuclear strand (green colour), and many scattered macronuclear nodules (grey colour). Distributions of speciation rates were obtained from MCMC simulations on the maximum clade credibility tree in the R-package diversitree. The y-axis represents posterior densities of speciation rates calculated on the basis of the MCMC simulations with exponential prior and 10,000 samples. Ninety-five per cent intervals of highest posterior density are indicated below densities.

The MRCAs of the orders Tracheliida, Dileptida, and the family Dileptidae had multiple micronuclei, while the MRCA of the family Dimacrocaryonidae exhibited only a single micronucleus (Fig. 4; Supplementary Table S2). This shift had no distinct effect on diversification because 52% of rhynchostomatians still possess multiple micronuclei and 48% of taxa have a single micronucleus. Also according to BiSSE analyses, speciation and extinction rates were equal for both states (multiple micronuclei vs. single micronucleus) in the best fitting model (Table 1; Supplementary Table S3). Likewise, the multi-state speciation and extinction analyses (MuSSE) did not recognise any significant difference between models where speciation rates for particular states of the macronuclear pattern were constrained to be equal and where they were allowed to vary (χ^2^ = 0.1132, df = 3, p* = *0.9902) (Supplementary Table S4). Nonetheless, the distribution of macronuclear patterns in extant taxa is rather variable, indicating some asymmetry especially in case of the compact macronucleus. Specifically, 38% of species exhibit two macronuclear nodules, 33% a moniliform strand, 21% many scattered nodules, and only 8% have a compact macronucleus^16^. In spite of this seeming unbalance among extant species, speciation rates of all four states strongly overlapped in MCMC simulations, where all speciation rates were allowed to vary (Fig. 4). This indicates that lineages with any state have similar probabilities to generate new species.

As concerns quantitative traits, a positive linear relationship between body size and diversification as well as between the relative proboscis length and diversification was preferred over other directional as well as constant and non-directional models analysed (Supplementary Table S5). Likelihood ratio test against the constant rate model supported these positive relationships for body length with χ^2^ = 5.4 (df = 2, p = 0.067) and for the relative proboscis length with χ^2^ = 5.1 (df = 2, p = 0.079).

## Discussion

### Asymmetry in diversification of rhynchostomatians

The class Litostomatea comprises a morphologically and ecologically diverse assemblage of free-living and endosymbiotic ciliates^10^. Bayesian relaxed molecular clock suggests that litostomateans emerged during the Cryogenian around 680 Mya and all fundamental litostomatean lineages diverged during the Paleozoic and Mesozoic periods^13^. Distribution of extant taxa is, however, rather uneven between the main free-living litostomatean lineages. According to the classification framework of Vďačný *et al*.^20^, there are over 250 species in the order Spathidiida^21^, more than 100 species in the order Pleurostomatida^22–26^, about 66 species in the Rhynchostomatia^14^, and only several dozens of species in the orders Haptorida, Didiniida, and Lacrymariida^24, 27, 28^. This cursory appraisal indicates that rhynchostomatians are not among the cladogenically most successful litostomateans. And, in addition, the two fundamental rhynchostomatian clades differ drastically in the number of known extant lineages. The order Tracheliida includes only two species, while the order Dileptida collates about 64 taxa^14^. Such a pronounced asymmetry has not been observed in any other litostomatean lineage, which provokes a question: what caused this conspicuous imbalance between fundamental rhynchostomatian clades?

Comparative analyses suggest that rhynchostomatians were caught in a plesiomorphic trap. In contrast to all other litostomateans, they still exhibit several ancient ciliate plesiomorphies, e.g., ventrally located cytostome, preoral kineties (~adoral membranelles) accompanying circumoral kinety (~paroral membrane), and formation of anarchic fields during morphogenesis^15, 16, 20, 29, 30^. In addition, the morphology of tracheliids has even a more plesiomorphic nature than that of dileptids and, hence, tracheliids are considered to be nearest in morphology to the putative last common progenitor of the Litostomatea^20, 29^. Specifically, tracheliids have an almost globular body with immobile proboscis whose dorsal side is distinctly shorter than the ventral one, their dorsal brush is not staggered, and their circumoral kinety is composed of dikinetids throughout. On the other hand, the last common ancestor of dileptids very likely possessed a narrow to almost rod-like body with agile proboscis whose ventral and dorsal sides are of a similar length, a staggered dorsal brush, and a hybrid circumoral kinety composed of dikinetids in the proboscis and of monokinetids around the cytostome^14, 15, 18^. We hypothesise that this suite of apomorphic features might be responsible for the pronounced cladogenic success of dileptids with respect to tracheliids. However, this hypothesis could not be directly tested with the BiSSE approach, because this technique is sensitive to strong tip ratio bias.

### Traits with symmetry in net-diversification rates

The present analyses indicated that the dorsal brush structure, contractile vacuole apparatus, and the number of extrusome types have an impact on asymmetry in the ability of particular rhynchostomatian lineages to diversify. On the other hand, particular states of the number of micronuclei and the macronucelar pattern did not differ in their net-diversification rates (Table 1; Supplementary Tables S3 and S4). This is not surprising since the nuclear apparatus is of a paramount significance for survival^31, 32^. Therefore, we speculate that all nuclear patterns are under similar selection pressure, which in turn causes that lineages with any state have similar probabilities to generate new species. The evolutionary significance of the nuclear apparatus in rhynchostomatians was recognised already by Dragesco^33^ and Jankowski^34^, who reflected this in their proposals of classification concepts in dileptids. Later on, most of their suggestions have been corroborated with the onset of molecular phylogenetic techniques^15–19^.

However, we cannot exclude that various nuclear character states have some indirect effect on asymmetry in net-diversification rates. Thus, different nuclear character states may have different impacts on fitness and may be also correlated with other characters recognised as causing asymmetries in the ability of particular rhynchostomatian lineages to diversify. To cast more light onto this problem, an experimental setting uncovering mutations that negatively or positively influence fitness of one or other of the studied character traits would be needed.

### Quantitative traits and diversification of rhynchostomatians

Quantitative traits have been long considered to affect speciation and extinction rates^35^, although the postulated hypotheses have been conflicting. Species with smaller bodies have been suggested to have higher speciation rates or lower extinction rates than species with larger bodies^36–38^. In contrast, Cope’s rule based on paleontological evidence has indicated that body size increases over evolutionary time, with descendent species tending to be larger than their ancestors^39, 40^. To overcome these problems, FitzJohn^41^ developed a likelihood-based method for testing hypotheses about quantitative state speciation and extinction. Using this approach we found support for increasing diversification in rhynchostomatians with growing body length and relative length of the proboscis (Supplementary Table S5). Indeed, rhynchostomatians are much larger than all other litostomateans. They are usually 150–500 μm long, with a maximum of 1,500 μm^14^. On the other hand, spathidiids are typically 60–150 μm long, with a maximum of 400 μm^21^ and pleurostomatids are mostly 50–200 μm long, with a maximum of 700 μm^22–26^. Since spathidiids and pleurostomatids are smaller but more diverse than rhynchostomatians, there must be clade-specific differences in diversification across litostomatean groups. Clade-specific diversification was also detected within primates, whereby elevation of speciation rates along with the increasing body size was concentrated only in the Cercopithecoidea and Hominoidea clades^41^. Taking into account the diversity and body size patterns of main litostomatean lineages, we hypothesise that large bodies increase the diversification of rhynchostomatians, but very likely through decreasing extinction.

### Perspectives

The diversity patterns of ciliates, a major group of microbial eukaryotes, have become a subject of numerous studies^9, 42–44^. On the other hand, intrinsic traits responsible for shaping species richness of particular ciliate groups are largely unknown. Understanding trait-dependent diversification and relationships between evolution of intrinsic and extrinsic traits is principal for sound interpretation of the patterns observed. Therefore, future workers investigating ciliate biodiversity should consider interlinking morphological data with molecular phylogenies. Moreover, our analyses uncovered an interesting trend, survival of some ciliate groups over several hundred million years could be associated with comparatively large and complex bodies. This combination, however, rather lowers extinction than promotes speciation. Our hypothesis could be in the future tested on the ancient, mostly large-bodied karyorelictean ciliates.

## Material and Methods

### Collection, sample processing and sequencing

During our long-term research we have collected 19 out of *ca*. 54 comparatively well known rhynchostomatian species from all over the world. There are about 10 further insufficiently described taxa whose identity is obscure^14^. To minimise bias owing to incomplete taxon sampling, numbers of taxa were held more or less proportional to the size of the two main dileptid lineages. Thus, we sampled six out of the 24 well-described species from the family Dileptidae and 11 out of the 30 well-known species from the family Dimacrocaryonidae. Since we cannot exclude that our sampling is unbiased, potentially type II errors (failing to reject the null hypothesis when the alternate hypothesis is true) can occur in BiSSE analyses. To avoid these errors, we utilised simplified models as recommended by Davis *et al*.^45^. Further, we used a Bayesian framework and corrections for missing taxa by specifying the fraction of sampled taxa having particular character states, as recommended by Silvestro *et al*.^46^ (for further details, see ‘Diversification analyses’ below).

Newly sequenced species were collected from various terrestrial and aquatic habitats (Supplementary Table S6). Terrestrial samples were processed using the non-flooded Petri dish method, while aquatic samples were examined directly after transportation to the laboratory. Taxonomic methods followed Vďačný & Foissner^14^. After species identification, several cells were picked, washed and transferred into 1.5 ml microtubes, where they were stored for molecular investigations. Genomic DNA of the Spanish population of *Rimaleptus binucleatus* was extracted as described by Vďačný & Rajter^18^, while DNA isolation of all other species/populations was according to Jang *et al*.^19^. The 18 S rRNA gene, the ITS1–5.8S-ITS2 region, and the D1D2 domain of the 28 S rRNA gene were amplified according to Vďačný *et al*.^16^, Vďačný & Rajter^18^, Jang *et al*.^19^, Medlin *et al*.^47^, and Sonnenberg *et al*.^48^. PCR products were directly sequenced on an ABI 3730 automatic sequencer (Macrogen Inc., Seoul, Korea), using PCR primers and an internal primer (5′-CCG CGG TAA TTC CAG CTC-3′). Sequence fragments from individual samples were checked and assembled into contigs using Geneious ver. 6.1.6 (Biomatters, http://www.geneious.com/).

### Tree-building methods

All available 18 S, ITS-region and D1D2 rhynchostomatian sequences were aligned and reliability of alignments was assessed on the GUIDANCE2 Server (http://guidance.tau.ac.il/ver2/), using the MAFFT algorithm and 100 bootstrap repeats^49^. The most appropriate nucleotide substitution models were chosen for each marker separately (Supplementary Table S7) with the help of the Akaike Information Criterion in jModelTest ver. 2.0.1^50^. Bayesian inference was run under the best fitting evolutionary models in MrBayes ver. 3.2.1^51^, using four independent chains, five million generations and a sample frequency of 1,000 on the CIPRES Portal ver. 1.15^52^. After reaching stationarity, posterior probabilities of a 50% majority-rule consensus tree were computed. The tree was *a posteriori* rooted according to our previous studies^14–18^, i.e. the root was placed on a branch separating the orders Tracheliida and Dileptida.

Apart from the Bayesian gene tree, a species tree was constructed using the Bayesian multispecies coalescent-based approach, as implemented in the program BEAST ver. 2.4.4^53^. This analysis was conducted using five datasets (18 S, ITS1, 5.8 S, ITS2, D1D2). The software BEAUti ver. 2.4.4 was used to create a *BEAST input file with the following settings: (i) GTR + I + Γ evolutionary model for the 18 S dataset, HKY + I + Γ model for the ITS1 dataset, TN93 + I model for the 5.8 S dataset, TN93 + Γ model for the ITS2 dataset, and GTR + Γ model for the D1D2 dataset; (ii) uncorrelated lognormal clock; and (iii) Yule process model for the species tree prior. MCMC analysis was run for 150,000,000 generations, sampling every 15,000^th^ generation. The convergence of all parameters to the stationary distribution in each run was inspected using the program Tracer ver. 1.6^54^. The final tree was summarized in TreeAnnotator ver. 2.4.4 using a 25% burn-in. The coalescent tree was rooted using the molecular clock technique, as implemented in the multispecies coalescent-based approach. The resulting position of the root was congruent with results from our previous phylogenetic analyses of rhynchostomatians, where outgroup taxa were used to root the tree^15–17^.

### Reconstruction of character evolution

We selected seven morphological characters that play a key role in classification of rhynchostomatians (Supplementary Table S8). Morphological data were obtained and scored according to our previous studies^14, 15, 18^. Character history was estimated for key nodes on the maximum clade credibility consensus tree, using the maximum-parsimony method as implemented in MESQUITE^55^ as well as on 100 trees from the posterior distribution of the BEAST coalescent analysis, using Bayesian and maximum-likelihood methods as implemented in BayesTraits ver. 1.0^56^.

### Diversification analyses

Diversification rates were estimated under pure-birth and birth-death models, using a Bayesian framework in the program BayesRate ver. 1.3.41^46^. This approach accommodates uncertainty in the inferred phylogeny, speciation and extinction-rate parameters, and missing species by specifying the proportion of taxa sampled. Fit of the models with and without extinction was assessed by Bayes factors, for which log marginal likelihoods were estimated over 100 trees from the posterior distribution of the BEAST coalescent analysis, using the thermodynamic integration option. Diversification-rate shifts were identified with maximum-likelihood technique, applied to the maximum clade credibility tree in MEDUSA^57^. This analysis also employed correction for incomplete taxon sampling and unsampled species were assigned to the extant clades according to Vďačný & Foissner^14^ and Vďačný & Rajter^18^.

Character-state associated diversification was estimated using the binary-state (BiSSE), multi-state (MuSSE), and qualitative trait (QuaSSE) methods. Since BiSSE and related methodologies are affected by the sample size and tip ratio bias, we minimised the potential of accepting the null hypothesis when the alternate hypothesis is true (type II error) by a combination of two strategies: (i) specification of the proportion of the taxa sampled for each character state and (ii) reduction of the number of model parameters. Our strategy followed Silvestro *et al*.^46^, who implemented a correction for missing taxa by specifying the fraction of sampled taxa having particular character states (Supplementary Table S9), and Davis *et al*.^45^, who recognised that simplified models can achieve greater power when tree size is small. Thus, as recommended by Davis *et al*.^45^, none of our tested BiSSE models had more than four free parameters.

BiSSE analyses were performed on dorsal brush, contractile vacuole and extrusome features in the program BayesRate. The Bayesian framework allows a more robust hypothesis testing, because it accounts for uncertainty in the inferred phylogeny as well as in the speciation and extinction-rate parameters. We tested four nested diversification models: (i) model 1 allowed both speciation and extinction rates to vary in both states; (ii) model 2 constrained speciation rates to be equal in both states but allowed extinction rates to vary; (iii) model 3 constrained extinction rates to be equal in both states but allowed speciation rates to vary; and (iv) model 4 constrained speciation and extinction rates to be equal in both states. Bayesian analyses were performed for each model over a sample of 100 trees from the posterior distribution of the BEAST coalescent analysis, running the MCMC simulation for 220 iterations of slice sampling per tree, discarding the first 20 samples per tree as burn-in. Corrections for incomplete taxon sampling were employed and diffuse priors were set for the diversification-rate parameters as suggested by Johnson *et al*.^7^. Fit of the models was assessed by Bayes factors, for which log marginal likelihoods were estimated over 100 trees from the posterior distribution, using the thermodynamic integration option. Dorsal brush pattern was not subjected to the BiSSE analyses because ordinary brush occurs only in two tracheliid species and staggered brush in all dileptids, which causes a strong tip ratio bias that could not be appropriately handled by BiSSE^45^.

MuSSE analyses were performed for macronuclear characteristics and QuaSSE analyses for body length and relative length of the proboscis, using the R-package diversitree^41, 58^. Maximum-likelihoods were estimated for several models, as specified in Supplementary Tables S4 and S5, on the maximum clade credibility consensus tree. We assessed the relative fit of the models to the data by calculating differences in AIC scores and AIC weights as well as by performing likelihood ratio test. Speciation rate of particular macronuclear characteristics was also subjected to Bayesian MCMC analysis with exponential prior and 10,000 samples.

### Data availability

Accession numbers of newly obtained DNA sequences are listed in Supplementary Table S6 and are available at https://www.ncbi.nlm.nih.gov/nucleotide/. Results of all analyses are included in this published article and its Supplementary information files. The datasets generated and/or analysed during the current study are available from the corresponding author on reasonable request.

## Electronic supplementary material

Supplementary material
