# Supplementary material for: Diversification dynamics of rhynchostomatian ciliates: the impact of seven intrinsic traits on speciation and extinction in a microbial group
Source: Sci Rep. 2017 Aug 30;7:9918. doi: 10.1038/s41598-017-09472-y (PMC5577237; doi:10.1038/s41598-017-09472-y)
Supplement: Supplementary file 1 — Supplementary material [file 41598_2017_9472_MOESM1_ESM.doc]

**Supplementary material**

**Diversification dynamics of rhynchostomatian ciliates: the impact of seven intrinsic traits on speciation and extinction in a microbial group**

**Peter Vďačný^1^, Ľubomír Rajter^1^, Shahed Uddin Ahmed Shazib^2^, Seok Won Jang^2^ & Mann Kyoon Shin^2,*^**

^1^Department of Zoology, Comenius University in Bratislava, Bratislava 84215, Slovak Republic. ^2^Department of Biological Sciences, University of Ulsan, Ulsan 44610, Korea. Correspondence and requests for materials should be addressed to P.V. (email: peter.vdacny@uniba.sk) or M.K.S. (email: mkshin@ulsan.ac.kr)

**Pages: 15**

**Figures: 1**

**Tables: 8**

**
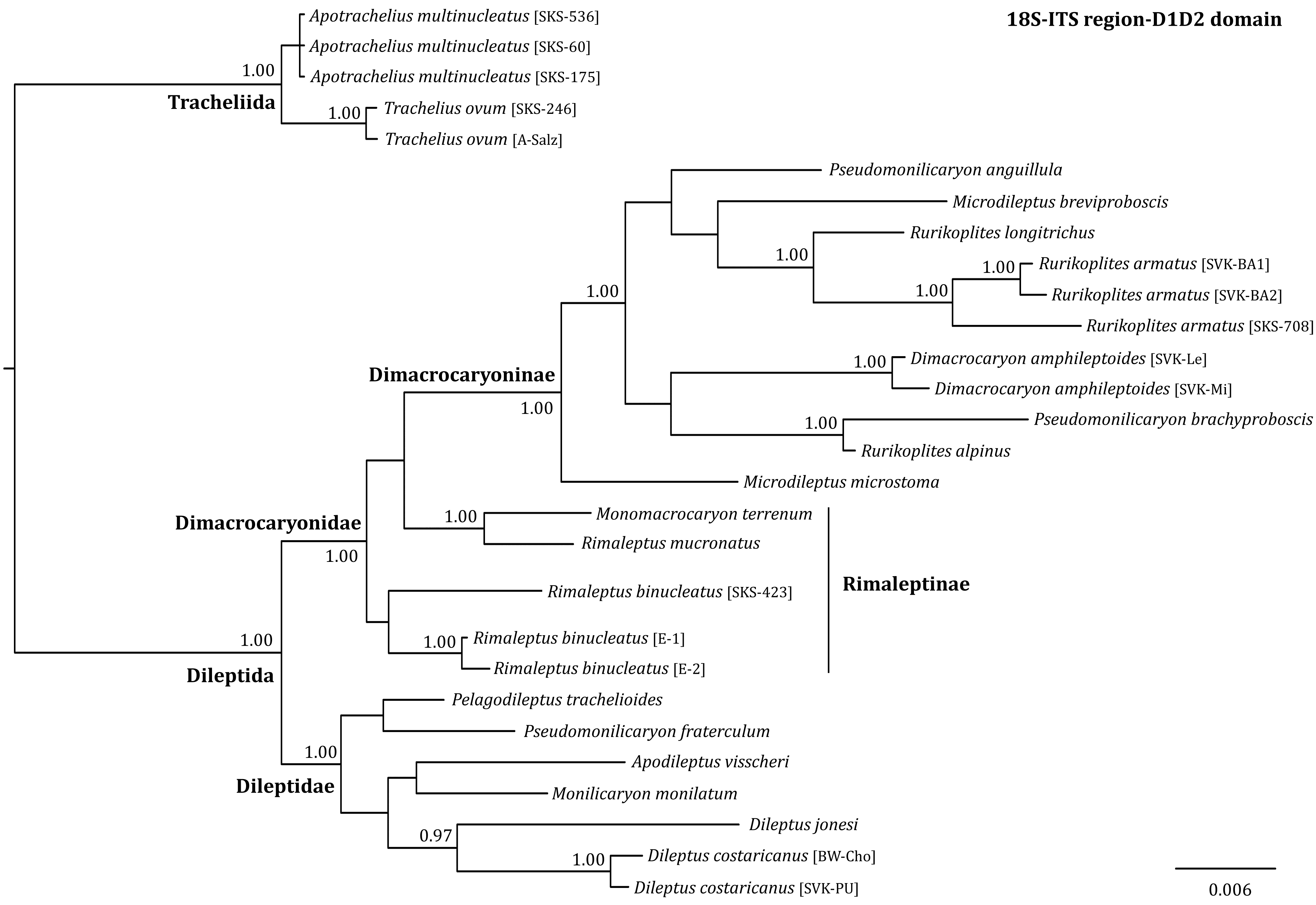
**

**Supplementary Figure S1.** Bayesian majority-rule consensus tree of the subclass Rhynchostomatia inferred from 18S rRNA gene, ITS-region, and D1D2 sequences. The scale bar indicates numbers of substitutions. Only posterior probabilities > 0.95 are shown.

**Supplementary Table S1.** Parameterisation and fitting of BayesRate models of diversification for rhynchostomatian ciliates. Tabulated are median rates of speciation (*λ*) and extinction (*μ*) as well as net diversification rate (*r*) and extinction fraction (*ε*) ± interquartile range estimated over 100 trees from the posterior distribution of the BEAST coalescent analysis.

| **Model** | **Group** | **logLik^1^** | **BF^2^** | ***λ*** | *μ* | ***r*** | ***ε*** |
| --- | --- | --- | --- | --- | --- | --- | --- |
| Pure-birth | Rhynchostomatia | 8.61 | 28.24 | 4.81810 ± 0.28040 | 0.00000 ± 0.0000 | 4.81810 ± 0.28040 | 0.00000 ± 0.00000 |
| Birth-death | Rhynchostomatia | 22.73 |  | 36.84018 ± 20.62533 | 33.05967 ± 20.80727 | 4.02778 ± 1.37330 | 0.89719 ± 0.07264 |
| **Clade specific rates under the birth-death model** | | | | | | | |
|  | Tracheliidae |  |  | 4.09531 ± 5.03460 | 1.33042 ± 3.53304 | 2.20201 ± 2.49155 | 0.42779 ± 0.45169 |
|  | Dileptidae |  |  | 18.83137 ± 18.71598 | 15.29568 ± 18.89108 | 3.80546 ± 1.60684 | 0.81560 ± 0.21201 |
|  | Rimaleptinae |  |  | 10.47815 ± 13.07365 | 7.11182 ± 13.09309 | 3.47234 ± 1.86517 | 0.69640 ± 0.38442 |
|  | Dimacrocaryoninae |  |  | 32.69034 ± 26.84031 | 29.14601 ± 26.94268 | 3.79707 ± 1.62728 | 0.89378 ± 0.10473 |

**Notes:**

**1:** Log likelihoods (logLik) are marginal likelihoods estimated across the sample of 100 trees for BayesRate models.

**2:** BayesFactor (BF) is expressed relative to the model of highest marginal likelihood. The more higher the BayesFactor, the more strongly supported is the best model. BF > 10 is interpreted as very strong support.

**Supplementary Table S2.** Ancestral state reconstruction for seven nodes of interest. Tabulated are presence (1) or absence (0) of character state under maximum parsimony (MP) as well as median probabilities under maximum likelihood (ML) and Bayesian inference (BI) ± the interquartile range across 100 trees from the posterior distribution of the BEAST coalescent analysis. For state coding, see Supplementary Table S8.

| **Node of interest** | **Characteristic** | **State** | **MP** | **ML** | **BI** |
| --- | --- | --- | --- | --- | --- |
| Root | Macronuclear pattern | A | 0 | 0.25690 ± 0.00389 | 0.25000 ± 0.01572 |
|  |  | B | 0 | 0.22016 ± 0.02125 | 0.25003 ± 0.02903 |
|  |  | C | 0 | 0.26137 ± 0.00969 | 0.25001 ± 0.01453 |
|  |  | D | 1 | 0.26191 ± 0.00816 | 0.25007 ± 0.01943 |
|  | Micronucleus, number | 0 | 0 | 0.06122 ± 0.02747 | 0.08637 ± 0.23528 |
|  |  | 1 | 1 | 0.93878 ± 0.02747 | 0.91363 ± 0.23528 |
|  | Extrusomes, number of types | 0 | 1 | 0.49926 ± 0.06954 | 0.49963 ± 0.03692 |
|  |  | 1 | 1 | 0.50074 ± 0.06954 | 0.50037 ± 0.03692 |
|  | Contractile vacuole pattern | 0 | 1 | 0.47427 ± 0.00980 | 0.48825 ± 0.03011 |
|  |  | 1 | 1 | 0.52573 ± 0.00980 | 0.51175 ± 0.03011 |
|  | Contractile vacuoles, number | 0 | 0 | 0.50000 ± 0.00000 | 0.37309 ± 0.33706 |
|  |  | 1 | 1 | 0.50000 ± 0.00000 | 0.62691 ± 0.33706 |
|  | Dorsal brush pattern | 0 | 1 | 0.34086 ± 0.03640 | 0.37058 ± 0.65888 |
|  |  | 1 | 1 | 0.65914 ± 0.03640 | 0.62942 ± 0.65888 |
|  | Brush rows, number | 0 | 1 | 0.98219 ± 0.00311 | 0.95600 ± 0.17238 |
|  |  | 1 | 0 | 0.01781 ± 0.00311 | 0.04400 ± 0.17238 |
| Tracheliida | Macronuclear pattern | A | 1 | 0.45783 ± 0.26701 | 0.48315 ± 0.17596 |
|  |  | B | 0 | 0.00203 ± 0.00210 | 0.00504 ± 0.02562 |
|  |  | C | 0 | 0.05846 ± 0.22039 | 0.03675 ± 0.09844 |
|  |  | D | 1 | 0.31232 ± 0.39190 | 0.45989 ± 0.32104 |
|  | Micronucleus, number | 0 | 0 | 0.00018 ± 0.00007 | 0.00026 ± 0.00120 |
|  |  | 1 | 1 | 0.99982 ± 0.00007 | 0.99974 ± 0.00120 |
|  | Extrusomes, number of types | 0 | 1 | 0.80194 ± 0.24630 | 0.79744 ± 0.23856 |
|  |  | 1 | 1 | 0.19806 ± 0.24630 | 0.20256 ± 0.23856 |
|  | Contractile vacuole pattern | 0 | 1 | 0.99100 ± 0.00413 | 0.98334 ± 0.06005 |
|  |  | 1 | 0 | 0.00900 ± 0.00413 | 0.01666 ± 0.06005 |
|  | Contractile vacuoles, number | 0 | 0 | 0.48732 ± 0.09777 | 0.00832 ± 0.04952 |
|  |  | 1 | 1 | 0.51268 ± 0.09777 | 0.99168 ± 0.04952 |
|  | Dorsal brush pattern | 0 | 1 | 0.99986 ± 0.00006 | 0.99999 ± 0.00038 |
|  |  | 1 | 0 | 0.00014 ± 0.00006 | 0.00001 ± 0.00038 |
|  | Brush rows, number | 0 | 1 | 0.99983 ± 0.00008 | 0.99961 ± 0.00204 |
|  |  | 1 | 0 | 0.00017 ± 0.00008 | 0.00039 ± 0.00204 |
| Dileptida | Macronuclear pattern | A | 0 | 0.34139 ± 0.11716 | 0.22914 ± 0.14802 |
|  |  | B | 1 | 0.26840 ± 0.06288 | 0.25724 ± 0.13080 |
|  |  | C | 0 | 0.19906 ± 0.11424 | 0.25008 ± 0.11703 |
|  |  | D | 1 | 0.21192 ± 0.03200 | 0.24998 ± 0.09929 |
|  | Micronucleus, number | 0 | 1 | 0.0000 ± 0.00000 | 0.21419 ± 0.19606 |
|  |  | 1 | 1 | 1.0000 ± 0.00000 | 0.78581 ± 0.19606 |
|  | Extrusomes, number of types | 0 | 1 | 0.50000 ± 0.00000 | 0.47354 ± 0.25748 |
|  |  | 1 | 1 | 0.50000 ± 0.00000 | 0.52646 ± 0.25748 |
|  | Contractile vacuole pattern | 0 | 1 | 0.28789 ± 0.04710 | 0.34487 ± 0.23627 |
|  |  | 1 | 1 | 0.71211 ± 0.04710 | 0.65513 ± 0.23627 |
|  | Contractile vacuoles, number | 0 | 0 | 0.50000 ± 0.00000 | 0.08921 ± 0.44369 |
|  |  | 1 | 1 | 0.50000 ± 0.00000 | 0.91079 ± 0.44369 |
|  | Dorsal brush pattern | 0 | 0 | 0.00867 ± 0.01640 | 0.00372 ± 0.01990 |
|  |  | 1 | 1 | 0.99133 ± 0.01640 | 0.99628 ± 0.01990 |
|  | Brush rows, number | 0 | 1 | 1.0000 ± 0.00000 | 0.98862 ± 0.04084 |
|  |  | 1 | 0 | 0.0000 ± 0.00000 | 0.01138 ± 0.04084 |
| Dileptidae | Macronuclear pattern | A | 0 | 0.28485 ± 0.04977 | 0.18398 ± 0.18155 |
|  |  | B | 0 | 0.01808 ± 0.00606 | 0.09699 ± 0.18432 |
|  |  | C | 1 | 0.35884 ± 0.06066 | 0.27727 ± 0.14058 |
|  |  | D | 1 | 0.33204 ± 0.03481 | 0.32133 ± 0.27825 |
|  | Micronucleus, number | 0 | 0 | 0.00040 ± 0.00032 | 0.00076 ± 0.00553 |
|  |  | 1 | 1 | 0.99960 ± 0.00032 | 0.99924 ± 0.00553 |
|  | Extrusomes, number of types | 0 | 0 | 0.44333 ± 0.55667 | 0.43342 ± 0.35078 |
|  |  | 1 | 1 | 0.55667 ± 0.40736 | 0.56658 ± 0.35078 |
|  | Contractile vacuole pattern | 0 | 1 | 0.51625 ± 0.04653 | 0.50025 ± 0.02087 |
|  |  | 1 | 1 | 0.48375 ± 0.04653 | 0.49975 ± 0.02087 |
|  | Contractile vacuoles, number | 0 | 0 | 0.50000 ± 0.00000 | 0.10570 ± 0.41169 |
|  |  | 1 | 1 | 0.50000 ± 0.00000 | 0.89430 ± 0.41169 |
|  | Dorsal brush pattern | 0 | 0 | 0.00029 ± 0.00025 | 0.00431 ± 0.03430 |
|  |  | 1 | 1 | 0.99971 ± 0.00025 | 0.99570 ± 0.03430 |
|  | Brush rows, number | 0 | 1 | 0.99965 ± 0.00029 | 0.99881 ± 0.01144 |
|  |  | 1 | 0 | 0.00036 ± 0.00029 | 0.00119 ± 0.01144 |
| Dimacrocaryonidae | Macronuclear pattern | A | 0 | 0.00886 ± 0.01238 | 0.05925 ± 0.15949 |
|  |  | B | 1 | 0.99003 ± 0.01095 | 0.87261 ± 0.32387 |
|  |  | C | 0 | 0.00034 ± 0.00172 | 0.02634 ± 0.13188 |
|  |  | D | 0 | 0.00064 ± 0.00086 | 0.01077 ± 0.10032 |
|  | Micronucleus, number | 0 | 1 | 0.99985 ± 0.00019 | 0.99973 ± 0.00110 |
|  |  | 1 | 0 | 0.00015 ± 0.00019 | 0.00027 ± 0.00110 |
|  | Extrusomes, number of types | 0 | 1 | 0.50001 ± 0.00044 | 0.48273 ± 0.07526 |
|  |  | 1 | 1 | 0.49999 ± 0.00044 | 0.51727 ± 0.07526 |
|  | Contractile vacuole pattern | 0 | 0 | 0.01885 ± 0.01725 | 0.04229 ± 0.22051 |
|  |  | 1 | 1 | 0.98005 ± 0.01725 | 0.95771 ± 0.22051 |
|  | Contractile vacuoles, number | 0 | 0 | 0.50000 ± 0.00032 | 0.06973 ± 0.29038 |
|  |  | 1 | 1 | 0.50000 ± 0.00032 | 0.93027 ± 0.29038 |
|  | Dorsal brush pattern | 0 | 0 | 0.00194 ± 0.00130 | 0.00062 ± 0.00425 |
|  |  | 1 | 1 | 0.99806 ± 0.00130 | 0.99938 ± 0.00425 |
|  | Brush rows, number | 0 | 1 | 0.0000 ± 0.00000 | 0.65592 ± 0.32198 |
|  |  | 1 | 1 | 1.0000 ± 0.00000 | 0.34408 ± 0.32198 |
| Rimaleptinae | Macronuclear pattern | A | 0 | 0.05706 ± 0.03203 | 0.21815 ± 0.18095 |
|  |  | B | 1 | 0.90533 ± 0.04661 | 0.53852 ± 0.45360 |
|  |  | C | 0 | 0.01413 ± 0.01407 | 0.10257 ± 0.20181 |
|  |  | D | 0 | 0.02247 ± 0.01385 | 0.07492 ± 0.20563 |
|  | Micronucleus, number | 0 | 1 | 0.99886 ± 0.00041 | 0.99672 ± 0.00980 |
|  |  | 1 | 0 | 0.00114 ± 0.00041 | 0.00328 ± 0.00980 |
|  | Extrusomes, number of types | 0 | 0 | 0.34074 ± 0.39749 | 0.31337 ± 0.37707 |
|  |  | 1 | 1 | 0.65926 ± 0.39749 | 0.68663 ± 0.37707 |
|  | Contractile vacuole pattern | 0 | 0 | 0.06283 ± 0.02670 | 0.12357 ± 0.30801 |
|  |  | 1 | 1 | 0.93717 ± 0.02670 | 0.87644 ± 0.30801 |
|  | Contractile vacuoles, number | 0 | 0 | 0.50000 ± 0.00033 | 0.38082 ± 0.14883 |
|  |  | 1 | 1 | 0.50000 ± 0.00033 | 0.61918 ± 0.14883 |
|  | Dorsal brush pattern | 0 | 0 | 0.00087 ± 0.00031 | 0.00745 ± 0.03480 |
|  |  | 1 | 1 | 0.99913 ± 0.00031 | 0.99255 ± 0.03480 |
|  | Brush rows, number | 0 | 1 | 0.99898 ± 0.00046 | 0.99702 ± 0.01481 |
|  |  | 1 | 0 | 0.00102 ± 0.00046 | 0.00298 ± 0.01481 |
| Dimacrocaryoninae | Macronuclear pattern | A | 0 | 0.00485 ± 0.00917 | 0.00693 ± 0.06312 |
|  |  | B | 1 | 0.99430 ± 0.00731 | 0.95920 ± 0.19314 |
|  |  | C | 0 | 0.00027 ± 0.00123 | 0.01316 ± 0.08657 |
|  |  | D | 0 | 0.00019 ± 0.00026 | 0.00246 ± 0.04253 |
|  | Micronucleus, number | 0 | 1 | 0.99990 ± 0.00015 | 0.99978 ± 0.00095 |
|  |  | 1 | 0 | 0.00010 ± 0.00015 | 0.00022 ± 0.00095 |
|  | Extrusomes, number of types | 0 | 1 | 0.68284 ± 0.38776 | 0.68354 ± 0.37707 |
|  |  | 1 | 0 | 0.31716 ± 0.38776 | 0.31646 ± 0.37707 |
|  | Contractile vacuole pattern | 0 | 0 | 0.07004 ± 0.06776 | 0.10312 ± 0.21490 |
|  |  | 1 | 1 | 0.92996 ± 0.06776 | 0.89688 ± 0.21490 |
|  | Contractile vacuoles, number | 0 | 0 | 0.50001 ± 0.00133 | 0.01692 ± 0.23815 |
|  |  | 1 | 1 | 0.49999 ± 0.00133 | 0.98308 ± 0.23815 |
|  | Dorsal brush pattern | 0 | 0 | 0.00008 ± 0.00012 | 0.00050 ± 0.00277 |
|  |  | 1 | 1 | 0.99992 ± 0.00012 | 0.99950 ± 0.00277 |
|  | Brush rows, number | 0 | 0 | 0.00009 ± 0.00015 | 0.00019 ± 0.00085 |
|  |  | 1 | 1 | 0.99991 ± 0.00014 | 0.99981 ± 0.00085 |

**Supplementary Table S3.** Parameterisation and fitting of BayesRate models for binary state diversification of rhynchostomatian ciliates. Tabulated are median rates of speciation (*λ*) and extinction (*μ*) as well as net diversification rates (*r*) and their differences (∆*r* = *r*_1_ – *r*_0_) ± interquartile range estimated over 100 trees from the posterior distribution of the BEAST coalescent analysis. Subscripts indicate character states as coded in Supplementary Table S8. BayesFactors (BF) are expressed relative to the model of highest marginal log likelihood (logLik). Best fitting models are in bold face.

| **Characteristic** | **Speciation** | **Extinction** | **logLik** | **BF** | ***λ*_0_** | ***λ*_1_** | *μ***_0_** | *μ***_1_** | ***r*_0_** | ***r*_1_** | **∆*r*** |
| --- | --- | --- | --- | --- | --- | --- | --- | --- | --- | --- | --- |
| Number of dorsal brush rows | free | free | 1.53 | 9.60 | 4.81 ± 11.43 | 4.48 ± 10.70 | 2.59 ± 10.60 | 2.40 ± 9.68 | 1.50 ± 1.61 | 1.45 ± 1.64 | 0.01 ± 1.57 |
|  | free | constrained | 4.44 | 3.78 | 6.23 ± 13.11 | 5.92 ± 12.87 | 4.00 ± 13.20 | 1.50 ± 0.97 | 1.22 ± 1.11 | –0.20 ± 0.85 | 0.71 ± 0.19 |
|  | **constrained** | **free** | **6.33** | **0.00** | **6.61 ± 16.95** | **6.61 ± 16.95** | **2.15 ± 5.94** | **1.51 ± 1.62** | **3.13 ± 9.82** | **1.12 ± 8.63** | **0.72 ± 0.48** |
|  | constrained | constrained | 4.48 | 3.70 | 5.86 ± 15.97 | 5.86 ± 15.97 | 3.64 ± 15.17 | 3.64 ± 15.17 | 1.48 ± 1.62 | 1.48 ± 1.62 | 0.00 ± 0.00 |
| Number of  extrusome types | free | free | –7.96 | 2.98 | 3.48 ± 7.50 | 5.61 ± 15.59 | 1.50 ± 6.23 | 3.28 ± 14.67 | 1.31 ± 1.61 | 1.49 ± 1.72 | 0.12 ± 1.73 |
|  | free | constrained | –7.20 | 1.46 | 5.80 ± 16.90 | 5.67 ± 16.16 | 3.70 ± 16.15 | 3.70 ± 16.15 | 1.38 ± 1.50 | 1.29 ± 1.63 | –0.06 ± 1.77 |
|  | **constrained** | **free** | **–6.47** | **0.00** | **6.27 ± 13.88** | **6.27 ± 13.88** | **4.00 ± 13.30** | **1.99 ± 5.39** | **1.55 ± 1.72** | **3.00 ± 7.59** | **1.01 ± 6.49** |
|  | constrained | constrained | –7.49 | 2.04 | 6.01 ± 15.11 | 6.01 ± 15.11 | 3.75 ± 14.15 | 3.75 ± 14.15 | 1.52 ± 1.73 | 1.52 ± 1.73 | 0.00 ± 0.00 |
| Number of  contractile  vacuoles | **free** | **free** | **–2.53** | **0.00** | **2.03 ± 4.09** | **6.14 ± 17.85** | **0.68 ± 2.43** | **3.79 ± 17.06** | **1.01 ± 1.61** | **1.55 ± 1.82** | **0.47 ± 1.78** |
|  | free | constrained | –3.73 | 2.40 | 5.06 ± 16.12 | 5.26 ± 15.29 | 3.04 ± 15.25 | 3.04 ± 15.25 | 1.34 ± 1.51 | 1.38 ± 1.70 | 0.06 ± 1.81 |
|  | constrained | free | –2.56 | 0.06 | 6.05 ± 12.73 | 6.05 ± 12.73 | 3.69 ± 12.16 | 1.89 ± 4.81 | 1.57 ± 1.70 | 2.85 ± 7.10 | 0.89 ± 6.09 |
|  | constrained | constrained | –3.38 | 1.70 | 6.08 ± 15.73 | 6.08 ± 15.73 | 3.75 ± 14.87 | 3.75 ± 14.87 | 1.57 ± 1.70 | 1.57 ± 1.70 | 0.00 ± 0.00 |
| Contractile  vacuole pattern | **free** | **free** | **–7.15** | **0.00** | **2.71 ± 4.79** | **5.80 ± 20.43** | **1.07 ± 3.55** | **3.54 ± 19.53** | **1.17 ± 1.47** | **1.54 ± 1.67** | **0.28 ± 1.71** |
|  | free | constrained | –7.72 | 1.14 | 5.55 ± 15.71 | 5.92 ± 16.47 | 3.73 ± 15.59 | 3.73 ± 15.59 | 1.10 ± 1.58 | 1.48 ± 1.64 | 0.35 ± 1.72 |
|  | constrained | free | –7.72 | 1.14 | 5.98 ± 15.84 | 5.98 ± 15.84 | 2.15 ± 6.23 | 3.72 ± 15.09 | 2.61 ± 7.19 | 1.48 ± 1.53 | –0.75 ± 6.13 |
|  | constrained | constrained | –7.34 | 0.38 | 5.81 ± 15.84 | 5.81 ± 15.84 | 3.67 ± 15.03 | 3.67 ± 15.03 | 1.46 ± 1.53 | 1.46 ± 1.53 | 0.00 ± 0.00 |
| Number of  micronuclei | free | free | 3.09 | 6.20 | 4.82 ± 13.02 | 4.18 ± 8.84 | 2.71 ± 12.04 | 2.08 ± 7.99 | 1.46 ± 1.67 | 1.38 ± 1.55 | –0.07 ± 1.60 |
|  | free | constrained | 5.83 | 0.72 | 5.95 ± 16.05 | 5.68 ± 15.31 | 3.74 ± 15.24 | 3.74 ± 15.24 | 1.53 ± 1.74 | 1.19 ± 1.58 | –0.24 ± 1.72 |
|  | constrained | free | 5.85 | 0.68 | 6.25 ± 12.85 | 6.25 ± 12.85 | 4.06 ± 12.28 | 2.20 ± 6.20 | 1.48 ± 1.64 | 2.66 ± 6.07 | 0.80 ± 5.05 |
|  | **constrained** | **constrained** | **6.19** | **0.00** | **5.68 ± 16.17** | **5.68 ± 16.17** | **3.45 ± 15.58** | **3.45 ± 15.58** | **1.54 ± 1.65** | **1.54 ± 1.65** | **0.00 ± 0.00** |

**Supplementary Table S4.** Fitting of two MuSSE models for macronucleus-dependent diversification of rhynchostomatian ciliates, as estimated on the 50%-majority rule consensus tree from the BEAST coalescent analysis.

| **Model** | ***n*** | **lnLik** | **AIC** | **χ^2^** | **p** |
| --- | --- | --- | --- | --- | --- |
| Speciation and extinction rates constrained to be equal in all states | 14 | 4.311 | 19.378 |  |  |
| Speciation rates allowed to vary and extinction rates constrained to be equal in all states | 17 | 4.368 | 25.265 | 0.1132 | 0.9902 |

**Notes:** *n*, number of free parameters of a model; lnLik, log likelihood; AIC, Akaike information criterion; χ^2^, chi-square; p, probability.

**Supplementary Table S5.** Fitting of QuaSSE models for relationship between body length and relative length of the proboscis and diversification of rhynchostomatian ciliates, as estimated on the 50%-majority rule consensus tree from the BEAST coalescent analysis. Best fitting models are in bold face.

| **Characteristic** | **Model** | ***n*** | **lnLik** | **AIC** | **∆AIC** | **AIC-weight** |
| --- | --- | --- | --- | --- | --- | --- |
| Body length [µm] | constant | 3 | –1022.9 | 2051.8 | 1.4 | 0.2215 |
|  | linear | 4 | –1022.6 | 2053.2 | 2.8 | 0.1100 |
|  | sigmoidal | 6 | –1022.6 | 2057.2 | 6.8 | 0.0149 |
|  | modal | 6 | –1022.8 | 2057.6 | 7.2 | 0.0122 |
|  | **drift.linear** | **5** | **–1020.2** | **2050.4** | **0.0** | **0.4461** |
|  | drift.sigmoidal | 7 | –1019.0 | 2052.1 | 1.7 | 0.1907 |
|  | drift.modal | 7 | –1022.8 | 2059.5 | 9.1 | 0.0047 |
| Relative proboscis length | constant | 3 | –50.206 | 106.41 | 1.1 | 0.2680 |
|  | linear | 4 | –50.091 | 108.18 | 2.8 | 0.1106 |
|  | sigmoidal | 6 | –49.852 | 111.7 | 6.3 | 0.0190 |
|  | modal | 6 | –49.773 | 111.55 | 6.2 | 0.0205 |
|  | **drift.linear** | **5** | **–47.680** | **105.36** | **0.0** | **0.4530** |
|  | drift.sigmoidal | 7 | –47.570 | 109.14 | 3.8 | 0.0684 |
|  | drift.modal | 7 | –47.694 | 109.39 | 4.0 | 0.0604 |

**Notes:** *n*, number of free parameters of a model; lnLik, log likelihood; AIC, Akaike information criterion; ΔAIC, difference in AIC scores between model and the overall best fit model.

**Supplementary Table S6.** Characterization of 18S rRNA gene and ITS region sequences of rhynchostomatian ciliates used in phylogenetic analyses. Newly obtained sequences are in bold.

| **Taxon** | **Collection site** | **18S rRNA gene** | | | **ITS1-5.8S-ITS2 region** | | |
| --- | --- | --- | --- | --- | --- | --- | --- |
|  |  | **Length (nt)** | **GC (%)** | **GenBank entry** | **Length (nt)** | **GC (%)** | **GenBank entry** |
| *Apodileptus visscheri rhabdoplites* Vďačný and Foissner, 2012 | Ephemeral pond in the Austrian Central Alps, Gross-glockner-Hochalpenstrasse, surroundings of the Hochtor, Austria (N47°06' E12°48') | 1640 | 42.32 | HM581678 | 362 | 37.02 | JX070869^2^ |
| *Apotrachelius multinucleatus* Vďačný, Al-Rasheid and Foissner in Vďačný and Foissner, 2012 [SKS-536] | Freshwater from the Seonsaemi pond, Seonheul-ri, Jocheon-eup, Jeju, Korea (N33°28'41'' E126°43'08'') | 1582 | 42.41 | **MF288143** | 360 | 30.83 | **MF288134** |
| *Apotrachelius multinucleatus* Vďačný, Al-Rasheid and Foissner in Vďačný and Foissner, 2012 [SKS-60]^1^ | Freshwater from Meonmulkkak wetland, Seonheul-ri, Jocheon-eup, Jeju-si, Jeju-do, Korea | 1538 | 42.33 | KJ680554 | 360 | 30.83 | **MF288141** |
| *Apotrachelius multinucleatus* Vďačný, Al-Rasheid and Foissner in Vďačný and Foissner, 2012 [SKS-175] | Freshwater from Galti pond in Ulsan grand park, Ulsan, Korea (N35°31'46'' E129°16'52'') | 1587 | 42.41 | **MF288147** | 360 | 30.83 | **MF288142** |
| *Dileptus costaricanus* Foissner, 1995 [BW-Cho] | Soil from the Chobe River floodplain, Kabolebole Peninsula, Botswana (S25°00' E17°50') | 1641 | 41.74 | HM581679 | 365 | 34.52 | JX0708682 |
| *Dileptus costaricanus* Foissner, 1995 [SVK-PU] | Terrestrial moss [*Brachythecium rutabulum* (Hedw.) Schimp.] from a floodplain forest in the surroundings of the village of Pusté Úľany, Slovakia (N48°13' E17°33') | 1554 | 41.70 | KP868765 | 3541 | 34.74 | KP8687732 |
| *Dileptus jonesi* Dragesco, 1963 | Leaf litter and soil from wetland near the Hambeak mountain, Gohan-eup, Jeongseon-gun, Gangwon-do, Korea (N37°12'17'' E128°54'20'') | 1592 | 42.40 | **MF288144** | 351 | 39.88 | **MF288135** |
| *Dimacrocaryon amphileptoides amphileptoides*  (Kahl, 1931) [SVK-Le] | Leaf litter from the town of Levoča, Slovakia (N49°00' E20°35') | 1521 | 42.74 | KP868766 | 349^3^ | 38.39 | KP868774^2^ |
| *Dimacrocaryon amphileptoides amphileptoides*  (Kahl, 1931) [SVK-Mi] | Leaf litter from a park in the village of Mikšová, Bytča, Slovakia (N49°11' E18°30') | 1553 | 42.50 | KP868767 | 330^3^ | 38.78 | KP868775^2^ |
| *Microdileptus breviproboscis* (Foissner, 1981) | Terrestrial moss (*Hypnum cupressiforme* Hedw.) from the surroundings of a student dormitory in Mlynská dolina, Bratislava, Slovakia (N48°09' E17°04') | 1558 | 42.75 | KP868768 | 349^3^ | 38.11 | KP868776^2^ |
| *Microdileptus microstoma* (Vďačný and Foissner, 2008) | Floodplain soil from Boise, Idaho, USA (N43°38'10'' W116°13'50'') | 1642 | 43.05 | HM581676 | 313^3^ | 36.10 | JX070866^2^ |
| *Monilicaryon monilatum* (Stokes, 1886) | Freshwater from the river basin of the Taehwa River, Taehwa-dong, Jung-gu, Ulsan, Korea | 1582 | 42.28 | KJ680550 | – | – | – |
| *Monomacrocaryon terrenum* (Foissner, 1981) | Soil from the outskirts of the town of Selker, Upper Austria, Austria (N48°23'32" E14°33'18") | 1639 | 42.46 | HM581674 | 368 | 35.05 | JX070864^2^ |
| *Pelagodileptus trachelioides* (Zacharias, 1894) | Freshwater from shore of Lake Biwa near the Lake Biwa Museum, Karasuma Peninsula, Shiga Prefecture, Japan (N35°04'21'' E135°56'22'') | 1608 | 41.85 | AB558117 | – | – | – |
| *Pseudomonilicaryon anguillula* (Kahl, 1931) | Decaying wood mass from the Hwangseong park, Hwangseong- dong, Gyeongju-si, Gyeongsangbuk-do, Korea (N35°51'31'' E129°12'53'') | 1525 | 42.49 | KJ680551 | 365 | 37.53 | **MF288136** |
| *Pseudomonilicaryon brachyproboscis* Vďačný and Foissner, 2008 | Terrestrial moss from the Devínska Kobyla Hill, Bratislava, Slovakia (N48°11' E16°58') | 1548 | 43.02 | KP868769 | 331^3^ | 41.08 | KP868777^2^ |
| *Pseudomonilicaryon fraterculum* Vďačný and Foissner, 2012 | Soil from a puddle in front of the Idaho Transportation Department Building, Boise, Idaho, USA (N43°38'10'' W116°13'50'') | 1640 | 42.32 | HM581677 | 363 | 33.61 | JX070867^2^ |
| *Rimaleptus binucleatus* (Kahl, 1931) [SKS-423] | Upper-soil layer covered with leaf-litter from Unhwa-ri, Onyang-eup, Ulju-gun, Ulsan, Korea (N35°23'50'' E129° 14'01'') | 1534 | 42.31 | KJ680552 | 365 | 36.71 | **MF288137** |
| *Rimaleptus binucleatus* (Kahl, 1931) [E-1] | Upper soil layer with plant material from a pine forest, C’an Picafort S’Albafera, Playa de Muro, Mallorca, Spain (N39°48'32" E03°07'06") | 1641 | 42.54 | **MF449475** | – | – | – |
| *Rimaleptus binucleatus* (Kahl, 1931) [E-2] | Upper soil layer with plant material from a pine forest, C’an Picafort S’Albafera, Playa de Muro, Mallorca, Spain (N39°48'32" E03°07'06") | 1642 | 42.57 | **MF449476** | – | – | – |
| *Rimaleptus mucronatus* (Penard, 1922) | Floodplain soil from Boise, Idaho, USA (N43°38'10'' W116°13'50'') | 1639 | 42.52 | HM581675 | 367 | 35.69 | JX070865^2^ |
| *Rurikoplites armatus* (Foissner and Schade in Foissner, 2000) [SVK-BA1] | Leaf litter from a poplar tree (*Populus alba* L.) in the surroundings of Comenius University in Mlynská dolina, Bratislava, Slovakia (N48°09' E17°04') | 1546 | 42.63 | KP868771 | 343^3^ | 39.36 | KP868778^2^ |
| *Rurikoplites armatus* (Foissner and Schade in Foissner, 2000) [SVK-BA2] | Terrestrial moss [*Brachythecium rutabulum* (Hedw.) Schimp.] from the surroundings of Comenius University in Mlynská dolina, Bratislava, Slovakia (N48°09' E17°04') | 1550 | 42.71 | KP868772 | 343^3^ | 39.06 | KP868779^2^ |
| *Rurikoplites armatus* (Foissner and Schade in Foissner, 2000) [SKS-708] | Bark of elm-like tree from the Haeinsa temple, Gaya-myeon, Hapcheon-gun, Gyeongsangnam-do, Korea (N35°47'54'' E128°05'52'') | 1592 | 42.52 | **MF288145** | 354 | 37.85 | **MF288138** |
| *Rurikoplites alpinus* (Kahl, 1931) | Leaf litter from a park in the village of Mikšová, Slovakia (N49°11' E18°30') | 1554 | 43.37 | KP868770 | – | – | – |
| *Rurikoplites longitrichus* (Vďačný and Foissner, 2008) | Leaf litter and soil from the Sangnasan mountain, Bi-ri, Heuksan-myeon, Sinan-gun, Jeollanam-do, Korea (N34°41'39'' E125°25'01'') | 1580 | 42.85 | **MF288146** | 362 | 35.91 | **MF288139** |
| *Trachelius ovum* (Ehrenberg, 1831) [A-Salz] | Freshwater from ephemeral University pond, Salzburg, Austria (N47°48'34" E13°03'18") | 1636 | 42.29 | HM581673 | 358 | 31.56 | JX070863^2^ |
| *Trachelius ovum* (Ehrenberg, 1831) [SKS-246] | Freshwater from the Jumgol reservoir, Mugeo-dong, Nam-gu, Ulsan, Korea | 1536 | 42.06 | KJ680553 | 358 | 31.56 | **MF288140** |

**Notes:**

**1:** This accession was originally designated as *Trachelius ovum* (Ehrenberg, 1831).

**2:** GenBank entries also include variably long 5′ end of the 28S rRNA gene.

**3:** Partial ITS1 sequences.

**Supplementary Table S7.** Characterisation and evolutionary models of the datasets analysed.

| **Characteristic** | **Dataset** | | | | |
| --- | --- | --- | --- | --- | --- |
|  | **18S** | **ITS1** | **5.8S** | **ITS2** | **D1D2** |
| No. of taxa | 28 | 23 | 23 | 23 | 14 |
| No. of char. | 1549 | 61 | 154 | 109 | 170 |
| Model | TIM2 + I + Γ | HKY + I + Γ | TrN + I | TrN + Γ | TPM1uf + Γ |
| A | 0.2958 | 0.4081 | 0.3217 | 0.3865 | 0.3912 |
| C | 0.1825 | 0.1784 | 0.1703 | 0.1817 | 0.1801 |
| G | 0.2403 | 0.0837 | 0.2149 | 0.1604 | 0.2053 |
| T | 0.2814 | 0.3298 | 0.2930 | 0.2714 | 0.2234 |
| ti/tv | – | 0.9272 | – | – | – |
| [AC] | 1.6832 | – | 1.0000 | 1.0000 | 1.0000 |
| [AG] | 3.2450 | – | 4.5273 | 1.6461 | 4.2656 |
| [AT] | 1.6832 | – | 1.0000 | 1.0000 | 2.9956 |
| [CG] | 1.0000 | – | 1.0000 | 1.0000 | 2.9956 |
| [CT] | 5.1526 | – | 7.4554 | 4.0944 | 4.2656 |
| [GT] | 1.0000 | – | 1.0000 | 1.0000 | 1.0000 |
| I | 0.7300 | 0.1820 | 0.8270 | – | – |
| Γ | 0.5170 | 1.1590 | – | 0.2610 | 0.1910 |

**Notes:** The best fitting evolutionary models were selected for each dataset under the Akaike information criterion in jModelTest. A, C, G, T, base frequencies; ti/tv, transition/transversion ratio; [AC], [AG], [AT], [CG], [CT], [GT], rate substitution matrices; I, proportion of invariable sites; Γ, gamma distribution shape parameter.

**Supplementary Table S8.** Characters, character states and their coding used for diversification analyses.

| **Species** | **Average body length [µm]** | **Proboscis, % of body length** | **Macronu-cleus^1^** | **Micronu-cleus, number^2^** | **Extrusomes, number of types^3^** | **Contractile vacuole pattern^4^** | **Contractile vacuoles, number^5^** | **Dorsal brush pattern^6^** | **Brush rows, number^7^** |
| --- | --- | --- | --- | --- | --- | --- | --- | --- | --- |
| *Trachelius ovum* | 350 | 39.3 | A | 1 | 0 | 0 | 1 | 0 | 0 |
| *Apodileptus visscheri* | 250 | 33.1 | D | 1 | 1 | 1 | 1 | 1 | 0 |
| *Apotrachelius multinucleatus* | 300 | 26.1 | D | 1 | – | 0 | 1 | 0 | 0 |
| *Dileptus costaricanus* | 280 | 31.9 | D | 1 | 1 | 0 | 1 | 1 | 0 |
| *Dileptus jonesi* | 450 | 33.0 | D | 1 | 1 | 1 | 1 | 1 | 0 |
| *Dimacrocaryon amphileptoides* | 200 | 30.5 | B | 0 | 0 | 0 | 1 | 1 | 1 |
| *Microdileptus breviproboscis* | 150 | 26.9 | B | 0 | 0 | 1 | 1 | 1 | 1 |
| *Microdileptus microstoma* | 170 | 20.4 | B | 0 | 0 | 1 | 1 | 1 | 1 |
| *Momonilacaryon monilatum* | 600 | 22.0 | C | 1 | 1 | 1 | 1 | 1 | 0 |
| *Monomacrocaryon terrennum* | 370 | 33.7 | A | 0 | 1 | 1 | 1 | 1 | 0 |
| *Pelagodileptus trachelioides* | 490 | 35.0 | C | 1 | 1 | 0 | 1 | 1 | 0 |
| *Pseudomonilicaryon anguillula* | 140 | 22.1 | C | 1 | 0 | 1 | 1 | 1 | 1 |
| *Pseudomonilicaryon brachychoproboscis* | 140 | 19.0 | C | 1 | 1 | 1 | 1 | 1 | 1 |
| *Pseudomonilicaryon fraterculum* | 600 | 54.6 | C | 1 | 1 | 0 | 1 | 1 | 0 |
| *Rimaleptus binucleatus* | 250 | 34.4 | B | 0 | 1 | 1 | 0 | 1 | 0 |
| *Rimaleptus mucronatus* | 350 | 30.3 | B | 0 | 1 | 1 | 1 | 1 | 0 |
| *Rurikoplites alpinus* | 110 | 29.7 | B | 0 | 1 | 1 | 0 | 1 | 1 |
| *Rurikoplites armatus* | 175 | 27.3 | B | 0 | 1 | 1 | 0 | 1 | 1 |
| *Rurikoplites longitrichus* | 210 | 36.9 | B | 0 | 0 | 0 | 1 | 1 | 1 |

**Notes:**

**1:** Mononodulate macronucleus coded A, binodulate coded B, multinodulate – moniliform strand coded C, multinodulate – scattered nodules coded D.

**2:** Number of micronuclei ≤ 2 coded 0, > 2 micronuclei coded 1.

**3:** One extrusome type coded 0, two extrusome types coded 1.

**4:** Contractile vacuoles arranged dorsally and ventrally coded 0, only dorsally coded 1.

**5:** Two contractile vacuoles coded 0, > 2 vacuoles coded 1.

**6:** Dorsal brush ordinary coded 0, staggered coded 1.

**7:** More than two brush rows coded 0, two brush rows coded 1.

**Supplementary Table S9.** Specification of the fraction of the taxa sampled with particular character states for BiSSE analyses in BayesRate.

| **Characteristic** | ***ρ*_0_** | ***ρ*_1_** |
| --- | --- | --- |
| Number of dorsal brush rows | 0.39 | 0.44 |
| Number of extrusome types | 0.64 | 0.34 |
| Number of contractile vacuoles | 0.42 | 0.41 |
| Contractile vacuole pattern | 0.54 | 0.36 |
| Number of micronuclei | 0.45 | 0.37 |

**Notes:** *ρ*_0_, fraction of sampled taxa having state 0; *ρ*_1_, fraction of sampled taxa having state 1. Subscripts indicate character states as coded in Supplementary Table S8.
